# ESBL *E. coli* Urosepsis Resulting in Endogenous Panophthalmitis Requiring Evisceration of the Eye in a Diabetic Patient

**DOI:** 10.1155/2015/897245

**Published:** 2015-08-17

**Authors:** Tejaswini Arunachala Murthy, Pradeep Rangappa, Sangeetha Rao, Karthik Rao

**Affiliations:** ^1^Intensive Care Unit, Columbia Asia Referral Hospital, Yeshwantpur, India; ^2^Department of Ophthalmology, Columbia Asia Referral Hospital, Yeshwantpur, India

## Abstract

A primary infection in a remote site resulting in vision threatening complications like panophthalmitis in a person who is not immune-compromised is rare. We report a case of endogenous bilateral bacterial endophthalmitis progressing into panophthalmitis in one eye requiring evisceration of that eye. A patient admitted with severe ESBL *E. coli* urosepsis was effectively treated with source control (bilateral DJ stenting for hydroureteronephrosis) and antibiotics. She was found to have features suggestive of bilateral conjunctivitis which progressed to panophthalmitis possibly due to delay in appropriate diagnosis and treatment. Diagnosis requires a very high index of suspicion if eye involvement is noted in a patient with features of bacteraemia and early intervention could possibly produce better outcomes. To our knowledge, this is the first case of ESBL *E. coli* urosepsis complicated by microbiologically confirmed panophthalmitis.

## 1. Introduction

Spontaneous bilateral endogenous endophthalmitis occurs as a complication of severe bacteraemia and is reported to be associated with urosepsis [1]. As the complaint, presentation, and treatment will be focussed on the primary source of sepsis, vision threatening complication such as endophthalmitis is overlooked by the treating physicians as conjunctivitis. Any delay in treatment can cause fatal visual complications and leave the patients blinded for life. The incidence of endophthalmitis progressing to panophthalmitis requiring evisceration as a complication of Gram negative sepsis is worthy to be noted. We report a case of severe urosepsis which was treated with source control and despite appropriate antibiotic cover progressed to bilateral endophthalmitis and further to panophthalmitis in the left eye in a span of <24 hrs requiring evisceration of the eye.

## 2. Case-Report

A 51-year-old known diabetic, hypertensive lady, presented with history of fever, generalized weakness, 10 episodes of loose stools for 1 day, and altered sensorium for a few hours to a nearby hospital where she was treated with Ceftriaxone injection and Levofloxacin injection for suspected urinary tract infection (UTI). Due to extreme drowsiness and worsening haemodynamics, she was shifted to our hospital for further management. On examination, she was arousable to deep pain, disoriented, and afebrile but had tachycardia (HR: 135 bpm) and hypotension (BP 80/42 mmHg), with markedly reduced urine output. She was aggressively resuscitated with intravenous fluid boluses and Inj Ertapenem 1 gm I.V OD was given within one hour of arrival in the ED (as per hospital antibiotic policy). Initial investigations revealed 15–20 pus cells in the urine examination and high serum creatinine (1.9 mmol/L). Ultrasound scan of the abdomen showed bilateral hydroureteronephrosis which was confirmed by a CT scan which also showed perinephric fat stranding. She underwent emergency bilateral DJ stenting within 6 hours of arrival at the hospital.

**Figure 1:**
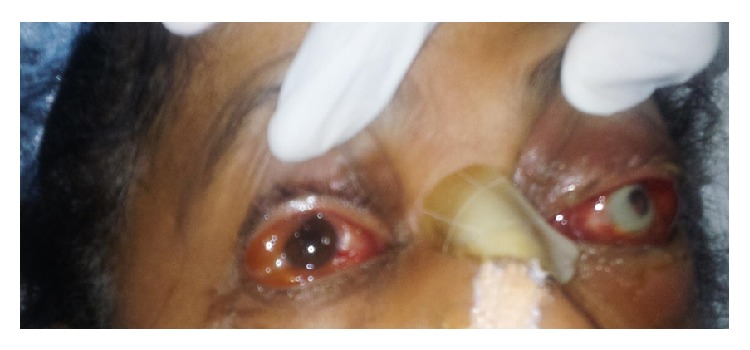
Bedside torch light examination showing corneal infiltrate in the left eye and conjunctival redness and suffusion in both eyes.

On the 3rd day of hospitalisation (3rd POD), it was noticed that she had redness of both eyes with severe left conjunctival suffusion. On examination, there was redness and watery discharge from both eyes (Figure 1). She was started on Ciprofloxacin eye drops and ophthalmology review was sought. On examination, she could count fingers at 1 metre distance in the right eye and had minimal perception of light in the left eye. Bedside torch light examination showed the following features:

 Right eye: anterior segment, normal lids, congested conjunctiva, clear cornea and normal depth anterior chamber.
 Left eye: tender oedematous lid, congested conjunctiva, corneal infiltrate present all round just inside the limbus, very shallow AC. and 4 mm pupil not reacting. The rest of the structures were not clear. Extraocular motility in the right eye showed mild restriction of all movements and complete restriction of all movements in the left eye. A preliminary diagnosis of left eye orbital cellulitis was made and poor prognosis was explained.

Further fundoscopic examination was deferred due to severe haemodynamic instability and lid edema of both eyes at this stage. The antibiotics were escalated to Moxifloxacin and Tobramycin eye drops. Next day the visual acuity worsened to perception of light in the right eye and no perception of light in the left eye. On examination, the right eye showed a pupillary membrane and the left eye showed a corneal infiltrate along the limbus and a vitreoretinal surgeon from a tertiary centre was asked to examine the patient for further management. CT scan of brain and orbit and Ultrasonography B (USG B) scan done confirmed the diagnosis of endophthalmitis in the right eye and panophthalmitis in the left eye.

USG B scan findings were as follows: vitreous cavity shows multiple dot-like echoes of low to moderate amplitude suggestive of vitreous debris. It also showed dome shaped membranous echoes of moderate to high amplitude suggestive of shallow choroidal detachments. Retina choroidal thickness was noted to be increased (2.33 mm).

**Figure 2:**
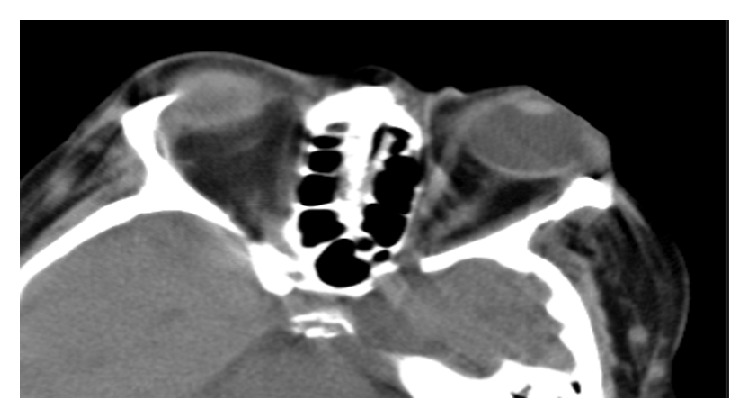
CT scan of the orbits showing bilateral preseptal soft tissue swelling, more on the left side, soft tissue stranding in left intra- and extraconal fat, and left-sided mild proptosis.

Brain and orbit CT revealed bilateral preseptal soft tissue swelling, more on the left side, soft tissue stranding in left intra- and extraconal fat, and left-sided mild proptosis (Figure 2). On the third day, the right eye findings had not changed and the left eye had worsening of periorbital edema and conjunctival suffusion and there were signs of corneal and scleral melt with exposed uveal tissue with an infiltrate.

**Figure 3:**
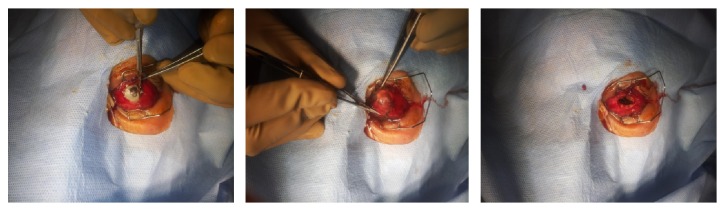
Intraoperative findings showed melting cornea and the left eye was eviscerated.

She was taken up for left eye evisceration surgery and the necrosed uveal tissue and vitreous humor were scooped out and sent for Gram stain, KOH mount bacterial and fungal culture, and histopathology (Figure 3). Culture of pus from the eviscerated left globe showed Extended Spectrum Beta Lactamase* Escherichia coli* (ESBL* E. coli*) which was sensitive to only Amikacin and carbapenems. Histopathology showed features consistent with ESBL E.coli panophthalmitis.

Intravitreal Amikacin,Vancomycin, andDexamethasone were injected into the right eye for endophthalmitis. There were signs of improvement in vision in the right eye with perception of hand movements. On examination, the lid edema had reduced, conjunctiva was normal, and cornea was clear. In view of this response, another dose of intravitreal injection of Amikacin, dexamethasone, and vancomycin was administered after 48 hours. The patient was discharged on the 6th day of admission during which time red glow of the fundus was seen and optic disc was faintly visible and follow-up after 6 months showed improvement in visual acuity to counting fingers at 6 metres' distance in the right eye.

## 3. Discussion

Endogenous bacterial endophthalmitis (EBE) is the result of bacterial multiplication within the eye after bacteria cross the blood-ocular barrier during bacteremia [2]. Worldwide, and in Asia, Gram negative infections (55%) are more frequent than Gram positive (45%) infections [3, 4]. However, some large retrospective studies outside Asia support the contrary [5, 6]. Endogenous endophthalmitis may account for 2–15% of all cases of endophthalmitis [5]. Underlying systemic illness and sometimes multiorgan dysfunction in the patient can cause diagnostic delay [5]. Some studies suggest a predilection to male preponderance with a relative risk ranging from 1.5 : 1 to 2 : 1 and right-sided ocular involvement in unilateral cases [1, 5]. A primary source of infection might remain unidentified in up to 40% of cases [5, 6].

The common etiologies of endophthalmitis and panophthalmitis are ocular trauma, surgery, and breach of blood-ocular barrier and systemic spread of bacteraemic and fungal showers [1, 5].

The incidence of sepsis causing endophthalmitis progressing very rapidly (<24 hours) to panophthalmitis is worthy to be noted. There is a case report of bilateral endogenous panophthalmitis in a patient with meningitis caused by streptococcal pneumonia but the culture from the eye tissue did not reveal the same bacteria to suggest endogenous spread to the eyes [2]. Various isolates amongst the Gram negative organisms worldwide have been of the* Haemophilus* and* Klebsiella* species [5, 7]. Data from the Indian subcontinent reveals fungal and polymicrobial contamination in several cases of traumatic endophthalmitis but Gram positive organisms predominate the picture in mixed (exogenous and endogenous) endophthalmitis population [8, 9]. In EBE, though one study reported Gram positive cocci as the commonest organism, Gram negative isolates were not negligible and resulted in fulminant infections supporting the need for multicentre trials to arrive at general conclusions [9]. A higher incidence of enteric Gram negative microorganisms causing EBE has been reported in many series with predominance of* Klebsiella* species (spp.) and* E. coli* worldwide [7, 9, 10]. Wong et al. reported the increased incidence of* Klebsiella* spp. EBE in the East Asian region [4, 9]. Urinary tract infection resulting in urosepsis in diabetic patients has been associated with severe endophthalmitis though* Klebsiella* remains the organism most frequently isolated [11, 12]. Irvine et al. reported an analysis on Gram negative organisms causing endophthalmitis and* Escherichia coli* (*E. coli*) emerged as the leading cause of the ocular infliction in the endogenous group in their study [13]. Walmsley et al. have highlighted the several case reports of EBE in urosepsis with* E. coli* being the most common organism [14].

Multi-drug resistant organisms causing endophthalmitis can affect outcomes and Extended Spectrum Beta Lactamase (ESBL) producing* E. coli* and* Klebsiella* in their endemic areas are emerging as the major causes for fulminant outcomes [5, 14]. They are exhibiting fluoroquinolone resistance and topical antibiotics (Moxifloxacin eye drops in our patient) could not have prevented the progression of infection. Even a diagnostic delay of a few hours in such cases might result in vision threatening complications.

The spread is commonly seen in immunocompromised patients and is rare in immunocompetent patients like our patient who had well-controlled diabetes (suggested by HbA1c of 6.1 at the time of admission) and hypertension. Transplant patients on immunosuppression, uncontrolled diabetics, and commonly insulin dependent diabetics and patients who are immunocompromised are among the most susceptible to breaching of the barrier and intraocular spread of infection [4, 14–16]. The time from onset of symptoms to presentation was about 3.5 days in a study by Schiedler et al. but in our patient a rapid progression was noted in a few hours [15]. The symptoms of endophthalmitis and panophthalmitis in septic patients can be overlooked as the treatment focus in a critical care setting would be to control the primary focus of infection. Since they can be rapidly progressive and can cause permanent threat to vision, a high index of suspicion and early ophthalmology review can prevent or reduce the severity of devastating complications like panophthalmitis. Any sign of worsening conjunctival suffusion or visual perception as told by the patient should be considered significant for further evaluation.

Endophthalmitis and panophthalmitis are best diagnosed by clinical examination supported by Ultrasound B scan and CT scan of the eyes. If the infection is limited to endophthalmitis or even early panophthalmitis, salvaging the vision can be tried with emergency vitrectomy and intravitreal antibiotics whereas panophthalmitis in later stages may require evisceration [16–18].

Response of endophthalmitis to treatment is often significant and improvement in vision correlates with the use of appropriate antibiotic. For endogenous ophthalmic infections, surgical management with emergency vitrectomy without any delay is recommended for patients who have no residual vision and may or may not continue to worsen with appropriate systemic, local, and intraocular antibiotics [16, 18]. Even a critical delay of a day can result in morbid outcomes. Early suspicion and diagnosis can prevent devastating and morbid surgeries.

## 4. Conclusion

Endogenous ocular infections have very high morbidity. Diabetic patients admitted to ICU with urosepsis might possibly benefit from a routine ophthalmology evaluation around the time of admission. With a high index of suspicion and by use of appropriate expertise assisted by imaging modalities, early interventions can have favourable outcomes and prevent devastating complications like blindness.
